# The Complete Mitochondrial Genome of *Plectorhinchus Chaetodonoides* (Perciformes: Haemulidae)

**DOI:** 10.1080/23802359.2022.2098852

**Published:** 2022-07-22

**Authors:** Haobin He, Guoqing Zhang, Ming Chen, Sen Yang, Guanyu Liang, Rishen Liang

**Affiliations:** a College of Animal Science and Technology, Zhongkai University of Agriculture and Engineering, Guangzhou, China; b Guangdong Provincial Water Environment and Aquatic Products Security Engineering Technology Research Center, Guangzhou, China; c Guangzhou Key Laboratory of Aquatic Animal Diseases and Waterfowl Breeding, Guangzhou, China

**Keywords:** Mitochondrial genome, *Plectorhinchus chaetodonoides*, phylogenetic analysis

## Abstract

*Plectorhinchus chaetodonoides* Lacepède, 1801 is a widespread multicolored sweetlips fish found in the Indo-West Pacific Ocean where its appearance and color patterns drastically change during growth. In this study, the whole mitochondrial genome of *P. chaetodonoides* was sequenced which revealed it is 16,546 bp long and contains 13 protein-coding genes, 22 transfer RNA (tRNA) genes, two ribosomal RNA (rRNA) genes, and one noncoding regulatory region. The GC content of the whole genome was 47.5% and 48.2%, 46.3%, 46.8%, 42.5% in the protein coding genes, tRNAs, rRNAs, and control regions, respectively. Molecular phylogenetic analysis resolved *P. chaetodonoides* as closely associated with *Diagramma pictum* and nested within a clade of Haemulidae that is allied with species from the Lutjanidae, Kyphosidae, Teraponidae, and Sciaenidae families. These results provide an essential genomic resource for future evolutionary and conservation studies of *P. chaetodonoides* as well as the Haemulidae family.

The Harlequin sweetlips fish, *Plectorhinchus chaetodonoides* Lacepède, 1801, belongs to the family Haemulidae of order Perciformes, and is a common multicolored sweetlips found in the Indo-Western Pacific Ocean. Adults are solitary and found near ledges or caves while juveniles have been discovered among corals. Morphologically, external appearance and color patterns in *P. chaetodonoides* could change drastically during its growth. Juveniles are brownish with large, well-defined creamy-white blotches on their bodies that later turn brown. Adults have a grayish background with large, deep-brown spots, which are quite distinct from the juvenile form (McKay 1984, 2001). Molecular sequences like mitochondrial DNA have been widely used in the species identification and phylogenetic relationship for the morphologically ambiguous species. In this study, the entire mitochondrial genome of *P. chaetodonoides* was sequenced, annotated, and analyzed in a phylogenetic context.

The Harlequin sweetlips *P. chaetodonoides* were collected in Sanya City, Hainan Province, China (18°24′05″N, 109°51′23″E) in August 2020 by setting nets. The sample was deposited in the laboratory of Zhongkai University of Agriculture and Engineering's College of Animal Science and Technology in Guangzhou, China (23°37′93″N, 113°45′15″E, voucher number: ZK-202008PC01, collector Information: Rishen Liang, cheetahliang@126.com). The total genomic DNA was extracted from muscle tissue using a DNA isolation kit (TIANGEN, Beijing, China). Sequencing of the mitogenome had been performed on the Illumina Novaseq 6000 platform and assembled using the de novo assembler SPAdes 3.11.0 (Dmitry et al. 2016). The average depth of sequencing for this mitogenome was 95.7X. Raw sequence reads were edited using NGS QC Tool kit (Patel and Jain 2012), the raw data totaled 4.78G, and the clean data totaled 4.64G, 2.93% raw reads were discarded. MITOS (http://mitos.bioinf.uni-leipzig.de/index.py) was used to annotate the whole mitochondrial genome. The proposed cloverleaf secondary structures of 22 tRNA genes were identified and drawn by tRNAscan-SE software (Lowe and Chan 2016). Organellar Genome DRAW v1.2 was used to create the circular genome map (Lohse et al. 2007).

**Figure 1.**
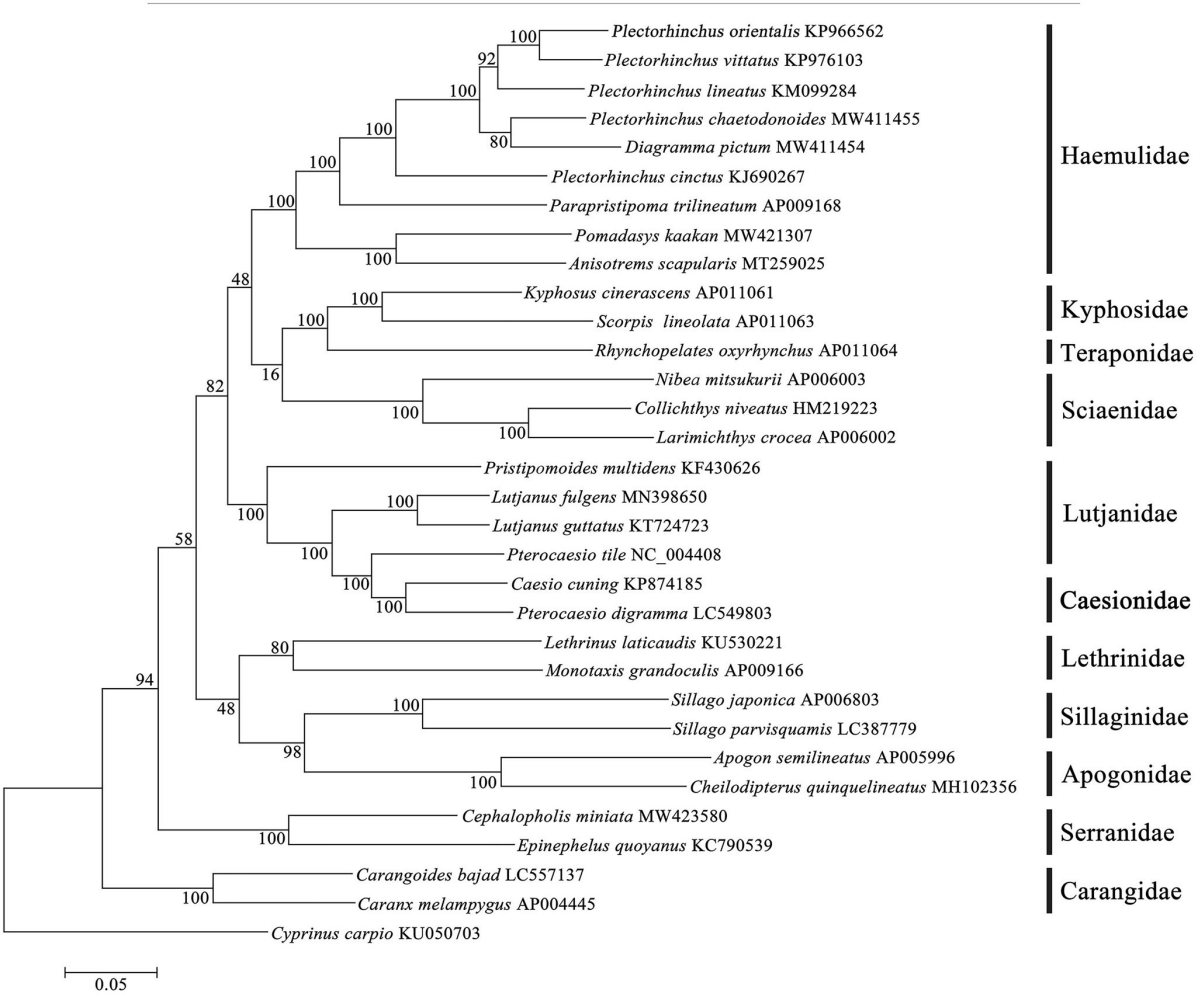
Molecular phylogenetic analysis based on maximum-likelihood analysis and 1000 bootstrap replicates of a data set of 12 protein-coding mitochondrial genes from *P. chaetodonoides* and 30 related Percoidei fishes, including nine Haemulidae species.

The complete mitogenome of *P. chaetodonoides* was 16,546 bp in length with a 47.5% GC content (GenBank accession number: MW411455) and contained 13 protein-coding genes, 22 transfer RNA genes (tRNAs), two ribosomal RNA (rRNA) genes, and one non-coding region. The GC content in the protein coding genes, tRNAs, rRNAs, and control regions was 48.2%, 46.3%, 46.8%, and 42.5%, respectively. Most genes were encoded on the heavy strand, except for the ND6 and eight tRNA genes (tRNA^Gln^, RNA^Ala^, RNA^Asn^, tRNA^Cys^, tRNA^Tyr^, tRNA^Ser^, tRNA^Glu^, and tRNA^Pro^), as observed in other vertebrates (Vandana et al. 2020; Luo et al. 2021; Zhang et al. 2021). Except for COI, which started with GTG, all protein-coding genes utilized ATG as a start codon. Various genes have different stop codon patterns: four genes ended with TAA (ND1, ATPase8, ND4L, and ND5), one ended with AGG (COI), the remaining genes ended with incomplete stop codons TA (ND2, ATPase6, COIII) or T: COII, ND3, ND4, ND6, and Cyt b.

A phylogenetic analysis using maximum-likelihood was conducted with data from the *P. chaetodonoides* mitogenome along with those from 30 other Percoidei fishes, including nine Haemulidae species, one Cypriniformes species *Cyprinus carpio* (DQ845759) was used for rooting. The concatenated sequences of 12 protein coding genes (except for ND6) were aligned in Clustal W (Thompson et al. 1994) and analyzed using MEGA version 7.0 software (Kumar et al. 2016). The substitution models for each12 protein coding genes were determined using jModelTest 2.1.5 (Darriba et al. 2012) and the optimal model GTR + G+I was selected. The phylogenetic tree was constructed using RAxML 8.0 software (Stamatakis 2014) with 1000 bootstrap replicates (Figure 1). The resulting phylogenetic reconstruction showed a monophyletic Haemulidae in which *P. chaetodonoides* is sister to *Diagramma pictum*, albeit with moderate bootstrap support. Although the backbone topology had low statistical support, Haemulidae forms a clade with Kyphosidae, Teraponidae, and Sciaenidae which is in turn sister to a clade containing Lutjanidae and Caesionidae. This mitochondrial genome provides an essential genomic resource for the conservation of *P. chaetodonoides* and further evolutionary studies of family Haemulidae.

## Data Availability

The genome sequence data that support the findings of this study are openly available in GenBank of NCBI at https://www.ncbi.nlm.nih.gov/ under the accession no. MW411455. The associated BioProject, SRA, and Bio-Sample numbers are PRJNA787728, SRR17190653, and SAMN23839731, respectively.
